# Neural Network Analysis for Microplastic Segmentation

**DOI:** 10.3390/s21217030

**Published:** 2021-10-23

**Authors:** Gwanghee Lee, Kyoungson Jhang

**Affiliations:** Department of Computer Science and Engineering, College of Engineering, Chungnam National University, Daejeon 34134, Korea; manggu251@gmail.com

**Keywords:** microplastic, neural network, segmentation, U-net, MultiResUNet, kernel weight histogram, tiny object segmentation

## Abstract

It is necessary to locate microplastic particles mixed with beach sand to be able to separate them. This paper illustrates a kernel weight histogram-based analytical process to determine an appropriate neural network to perform tiny object segmentation on photos of sand with a few microplastic particles. U-net and MultiResUNet are explored as target networks. However, based on our observation of kernel weight histograms, visualized using TensorBoard, the initial encoder stages of U-net and MultiResUNet are useful for capturing small features, whereas the later encoder stages are not useful for capturing small features. Therefore, we derived reduced versions of U-net and MultiResUNet, such as Half U-net, Half MultiResUNet, and Quarter MultiResUNet. From the experiment, we observed that Half MultiResUNet displayed the best average recall-weighted F1 score (40%) and recall-weighted mIoU (26%) and Quarter MultiResUNet the second best average recall-weighted F1 score and recall-weighted mIoU for our microplastic dataset. They also require 1/5 or less floating point operations and 1/50 or a smaller number of parameters over U-net and MultiResUNet.

## 1. Introduction

A neural network finding the location of tiny objects in an image can be used in a variety of fields, including disaster relief work to find victims from drone images taken on the mountain, commercial work to find cracks in products, military work to determine the condition of targets with satellites, public interest to find and remove microplastics in beach sand, etc. Among them, our research aims to solve the problem of finding microplastics in beach sand.

Microplastic is a plastic piece of 5mm or less [1]. These small pieces of plastic are mistaken for food and are consumed by coral, plankton, sea urchin, lobster, and fish. It reduces the reproduction, growth, and regeneration ability of marine life [2,3]. This problem accelerates and spreads to all ecosystems by the food chain, and humans are no exception.

Research related to microplastics was mainly aimed at making statistical data by identifying the types and numbers of microplastics that have been completely separated from seawater or sand. For example, a study was conducted to accurately identify the types of microplastics isolated from sand or sea water using precision equipment, such as microscopes, electron microscopes, etc. [4]. In addition, a machine learning technique was used to determine the type and number of microplastics isolated from beach sand [5]. In another paper [6], segmentation was performed on images of microplastics separated from sand using U-net [7]. In the same paper, VGG16 [8] was used to classify each microplastic type (fragments, pellets, or lines) in a given microplastic image. This paper introduces a study to explore a deep learning model that can be used to recognize and separate microplastics when sand scooped up from the beach is placed on a conveyor belt.

At first, U-net and MultiResUNet were explored as target networks to recognize microplastic in sand photos. Based on our observation of kernel weight histograms visualized using TensorBoard [9], it can be found that the later encoder stages of the two networks are not useful for capturing small features. Therefore, we derived reduced versions of U-net and MultiResUNet, such as Half U-net, Half MultiResUNet, and Quarter MultiResUNet. With experiments, we observed that Half MultiResUNet displayed the best average recall-weighted F1 score (r.w. F1 score) and recall-weighted mIoU (r.w. mIoU) and Quarter MultiResUNet the second best average r.w. F1 score and r.w. mIoU for our microplastic dataset. They also require very low floating point operations and much lower number of parameters over U-net and MultiResUNet. The five neural network architectures including the derived versions are explained in Section 4.

The contribution of this paper can be summarized as follows:

- Kernel weights histogram shows the distribution of weight values of each layer. By using this histogram, the neural network designer can determine the degree of layer utilization, and by classifying unnecessary layers according to the layer utilization degree, the neural network structure can be optimized. This paper demonstrates this optimization process;
- A neural network suitable for tiny object segmentation was proposed by deriving from the existing NNs (Neural Networks), and it shows better performance with only 10–20% of the amount of computation over the existing NNs.

The rest of this paper is organized as follows: Section 2 introduces related works and explains why some models have been employed and other models have not been used for experiments and analysis. Section 3 briefly describes the structures and characteristics of the five neural networks, i.e., two existing NNs and the three derived versions of NNs. Section 4 introduces recall-weighted metrics, such as r.w. F1 score, and r.w. mIoU, and the experimental settings, as well as the comparison between the segmentation performances of the five NNs, followed by a few insights that were obtained from the observation of the weight histogram. We have summarized and concluded the paper in Section 5.

## 2. Related Works

The task of locating an object in an image can be performed by detection or segmentation. However, the problem of finding the location of microplastics in sand photos is a problem of finding very small objects. Therefore, segmentation that predicts objects and backgrounds in units of pixels is more effective than detection that finds a location in the form of a bounding box because bounding boxes are not accurate at locating microplastics in pixel level. Segmentation can be divided into two main types of problems. Namely, semantic segmentation and instance segmentation. Semantic segmentation only needs to classify the class to which each pixel belongs. Recent semantic segmentation networks include GALDNet [10] and HANet [11]. However, in instance segmentation, objects belonging to the same class must also be distinguished from each other if they belong to different instances. Recent instance segmentation networks include EfficientPS [12] and Panoptic-DeepLab [13]. For the problem of finding the location of microplastics, semantic segmentation is suitable because it is not necessary to distinguish the identity of each object. However, neural network models for semantic segmentation, such as GALDNet and HANet, have been studied to recognize mid-large objects by atrous spatial pyramid pooling (ASPP) [14,15] and pyramid pooling [16]. Since our task is to recognize tiny objects, we had to search for models suitable for segmentation of small as well as large objects. Biomedical segmentation [7,17] has similar characteristics in a sense that it often needs to recognize even small objects. U-net or similar neural networks are employed for biomedical segmentation, to recognize cell boundaries and cancer regions. U-net appears to be a candidate NN for microplastic segmentation because it deals with small, as well as large objects with no regular shape. SegNet [17] is similar to, but simpler than U-net since it has a structure similar to that of a skip connection in U-net that passes the pooling index to the appropriate decoder stage. Although we trained the SegNet model using the microplastic dataset, all prediction results by the model came out as 0, which means that no training was completed at all. So, we excluded SegNet from our comparative study.

MultiResUNet [18] is also considered as a NN for microplastic segmentation because it exhibits better segmentation performance than U-net. MultiResUNet was derived from U-net, by replacing the skip connection with the residual path, and each encoder/decoder stage consists of MultiRes blocks with a residual block structure of ResNet [8]. The encoder stage means an embedding connection in which the width and height of the tensor are reduced either through pooling or by using a stride greater than 1 in the convolutional layer. The decoder stage means an expanding connection in which the width and height of the tensor are enlarged using upsampling or deconvolution.

Although DeepLab [14,19,20] exhibited satisfactory performance for middle- and large-sized objects because it employs ASPP, we excluded DeepLab because it has not been used actively in the segmentation of small or tiny objects.

## 3. Neural Networks for Microplastic Segmentation

**Figure 1:**
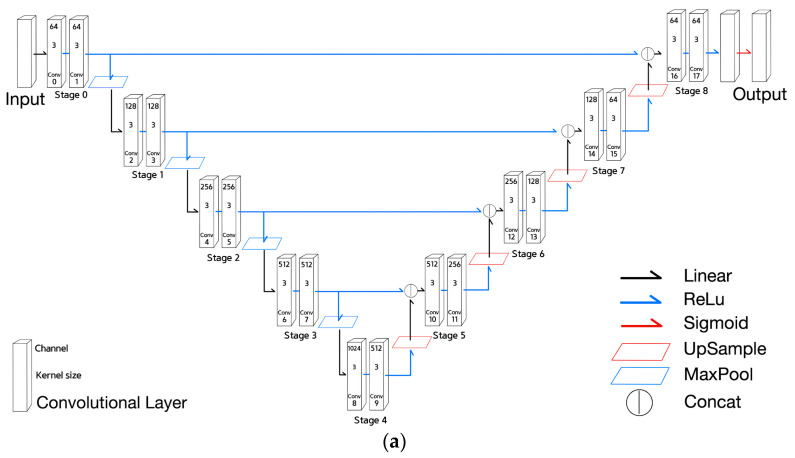

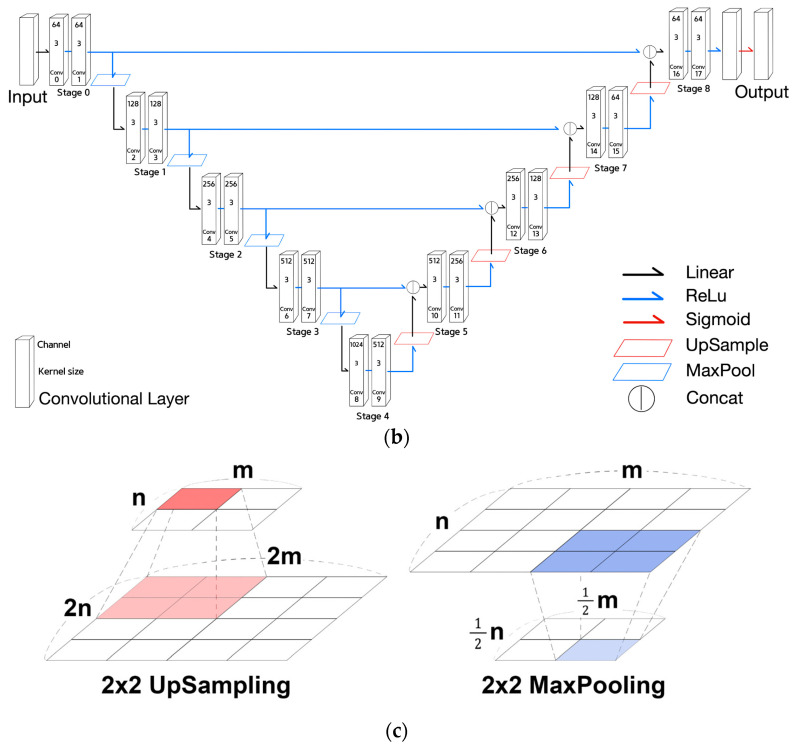
U-net and operations. (**a**) The architecture of U-net. (**b**) Graphs of linear, ReLU, and sigmoid activation functions. (**c**) Processes of upsampling and maxpooling.

This section briefly introduces the two existing NNs and the three derived versions of NNs used in microplastic segmentation. The first NN is U-net shown in Figure 1a. U-net consists of encoder stages, which are followed by decoder stages, as shown in Figure 1a. The dimensions of the output of each encoder stage are scaled down using a max pooling layer [21]. Each decoder stage accepts the output from the previous stage, which is upsampled with nearest interpolation [22]. The skip connection is used to maintain the positional information of the features by connecting the output of the encoder stage with the corresponding decoder stage. With respect to the aforementioned facts, the former half of the encoder stages combined with the corresponding decoder stages appear to include the characteristics required for determining small input features, while the latter half of the encoder stages include the characteristics required for capturing large input features. Because this study deals with minuscule objects, such as microplastics, we introduced a simplified U-net, called Half U-net, that contains the first half of the encoder stages along with the corresponding decoder stages. As shown in Figure 1b, ‘Linear’ Activation means that the input X is transferred to output Y as it is. ‘ReLU’ Activation is the same as ‘Linear’ Activation if the input X is greater than or equal to 0, and if the input value is less than 0, output becomes 0. ‘Sigmoid’ Activation converts the input into the continuous output value between 0 and 1 based on the exponential function. As shown in Figure 1c, upsampling expands the input 2 times in horizontal and vertical direction while maxpooling shrinks the input 1/2 times in both directions.

Equations (1)–(6) are used to describe the functionality of U-net.

- Equation (1) is the convolutional layer expression, where k and s are the kernel size and the stride of the kernel. In addition, the term l means the function applied to each layer, and the terms i and j the horizontal and vertical position of an element of a layer output. The term $K_{ij}^{l}$ means (i,j)-th kernel matrix of l layer, and the position of matrix element is expressed as [n,m]. The term $b_{ij}^{l}$ means (i,j)-th bias of l layer. The term n means a row position of a kernel, and the term m a column position of the kernel;
- Equation (2) is an expression of 2x2 MaxPooling. The result of $max^{2}{\left(X\right)}_{ij}^{l}$ is the maximum value among the 2x2 submatrix with its upper left position (2i,2j) of the matrix $X^{l}$;
- Equation (3) is an expression of 2x2 UpSampling. The result of positions (2i,2j) to (2i+1,2j+1) in the matrix $upsample^{2}{\left(X\right)}_{}^{l}$ is set to the $X_{ij}^{l}$ value;
- The stages of U-net can be expressed by Equation (4) to (6):
- Equation (4) is the equation of the 0th stage that indicates the application of two convolutional layers with kernel size of three on the input I and then ReLU(x) activation function expressed by max(0,x) is applied. The term I means an input tensor of a model;
- Equation (5) is the encoder stage equation that indicates the application of maxpooling to the previous stage, followed by two convolutional layers and then final ReLU activation function. The term $max^{2}$ used in this equation means 2x2 maxpooling;
- Equation (6) is the decoder stage equation. The equation means the concatenation of the corresponding encoder stage output with the upsampled previous stage output, followed by two convolutional layers and then the final ReLU activation. The term $upsample^{2}$ used in Equation (6) means 2x2 upsampling.
  (1) $$Conv^{k,s}{\left(X\right)}_{ij}^{l}=\overset{k}{\underset{n=0}{\sum }}\overset{k}{\underset{m=0}{\sum }}X\left[is+n,js+m\right]\circ K_{ij}^{l}\left[n,m\right]+b_{ij}^{l}$$
  (2) $$max^{2}{\left(X\right)}_{ij}^{l}=max\left({\left\{{\left\{X_{nm}^{l}\right\}}_{m=2j}^{2j+1}\right\}}_{n=2i}^{2i+1}\right)$$
  (3) $${\left\{{\left\{upsample^{2}{\left(X\right)}_{nm}^{l}\right\}}_{m=2j}^{2j+1}\right\}}_{n=2i}^{2i+1}=X_{ij}^{l}$$
  (4) $$Out_{0}^{stage}=max(0,Conv^{3,1}\left(Conv^{3,1}\left(I\right)\right))$$
  (5) $$Out_{\left\{1,2,3,4\right\}}^{stage}=max(0,Conv^{3,1}\left(Conv^{3,1}(max^{2}\left(Out_{i-1}^{stage}\right)\right)))$$
  (6) $$Out_{\left\{5,6,7,8\right\}}^{stage}=max(0,Conv^{3,1}\left(Conv^{3,1}\left(concat\left(Out_{8-i}^{stage},upsample^{2}\left(Out_{i-1}^{stage}\right)\right)\right)\right)))$$

**Figure 2:**
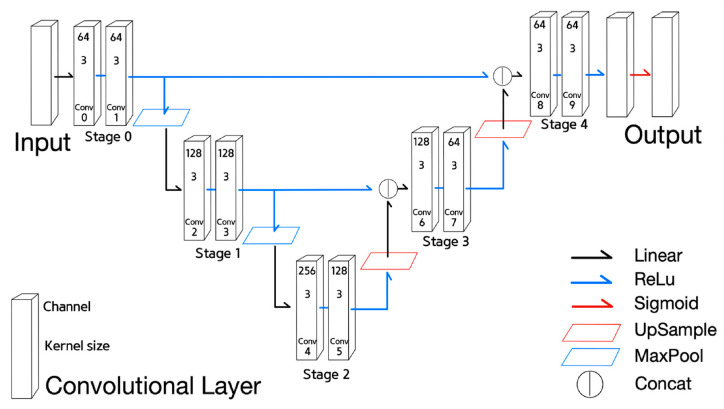
Half U-net.

Next is a derived NN from U-net, called Half U-net, shown in Figure 2. Half U-net is a simplified version of U-net, and contains only the 0th and 1st encoder stages along with their corresponding decoder stages. The fourth stage in U-net was also retained in Half U-net; however, with a reduced number of channels in the convolution layers.

Equations (7)–(9) are used to describe the functionality of Half U-net.

- States of Half U-net can be described in Equations (7)–(9);
- Equations (7)–(9) are defined similarly to Equations (4)–(6) of U-net, respectively. However, Equation (8) is for the encoder stage corresponding to the 1st or the 2nd stage and Equation (9) for the decoder stage corresponding to the 3rd and the 4th stage.

(7) $$Out_{0}^{stage}=max(0,Conv^{3,1}\left(Conv^{3,1}\left(I\right)\right))$$

(8) $$Out_{\left\{1,2\right\}}^{stage}=max(0,Conv^{3,1}\left(Conv^{3,1}(max^{2}\left(Out_{i-1}^{stage}\right)\right)))$$

(9) $$Out_{\left\{3,4\right\}}^{stage}=max(0,Conv^{3,1}\left(Conv^{3,1}\left(concat\left(Out_{4-i}^{stage},upsample^{2}\left(Out_{i-1}^{stage}\right)\right)\right)\right)))$$

**Figure 3:**
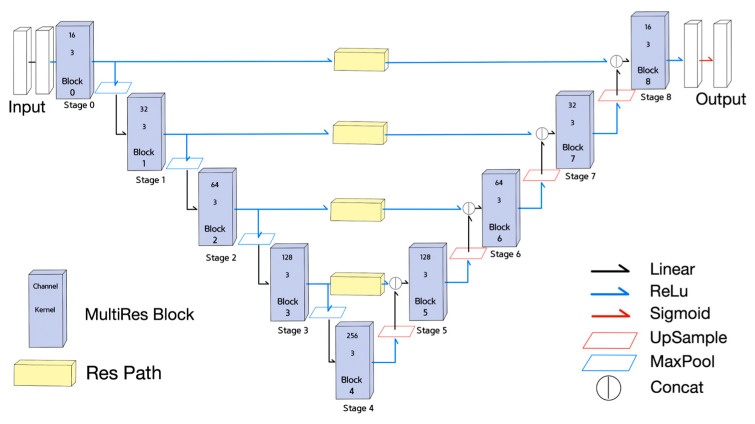
MultiResUNet.

**Figure 4:**
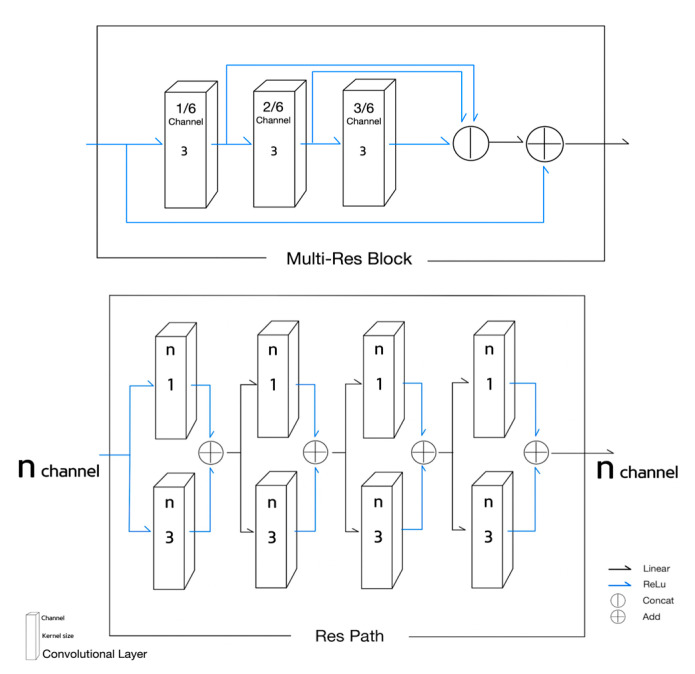
MultiRes block and residual path in MultiResUNet.

The third NN used for the experiment was the MultiResUNet [18], as shown in Figure 3. Each stage in U-net was replaced with a MultiRes block, and each skip connection was replaced with a residual path. The MultiRes block and the residual path illustrated in Figure 4 contain residual connections.

**Figure 5:**
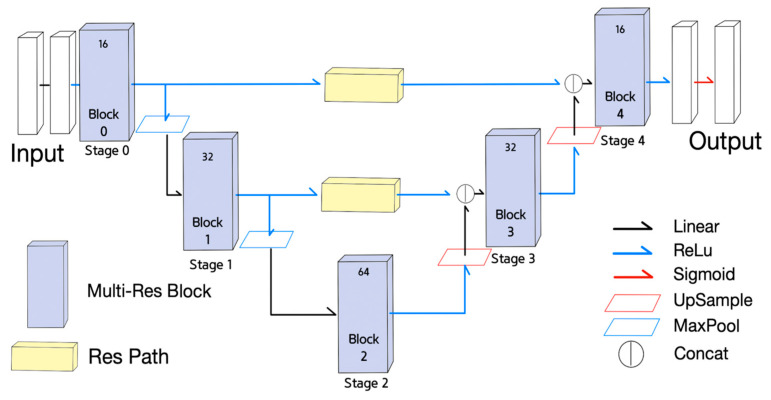
Half MultiResUNet.

The fourth neural network is a derived NN from MultiResUNet, called Half MultiResUNet, which is a simplified version of the MultiResUNet. Half MultiResUNet, shown in Figure 5, only claims the 0th and 1st encoder MultiRes blocks, and the 7th and 8th decoder MultiRes blocks, along with the 4th MultiRes block equipped with fewer channels than MultiResUNet. Half MultiResUNet seems to focus on the small features of the objects.

**Figure 6:**
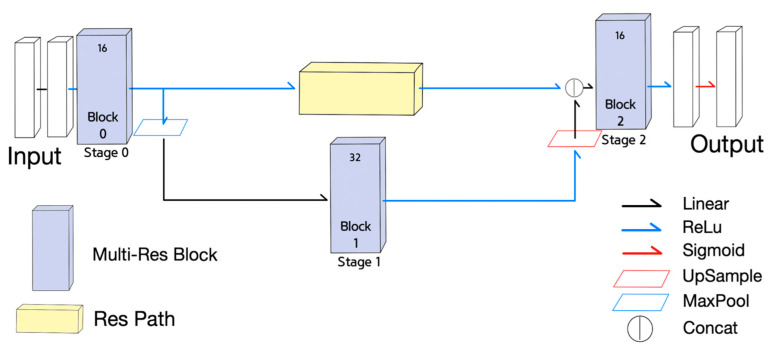
Quarter MultiResUNet.

The final NN is another derived NN from MultiResUNet, called Quarter MultiResUNet with only one encoder decoder block pair, which is the most simplified version of the MultiResUNet. Quarter MultiResUNet, shown in Figure 6, claims only the 0th and 8th MultiRes blocks, with the 4th MultiRes block containing fewer channels than MultiResUNet. Quarter MultiResUNet seems to focus on the segmentation of minuscule features of objects.

Equations (10)–(18) are used to describe the functionality of MultiResUNet.

- Equations (10)–(13) is for the MultiRes Block (MRB);
- The Equations (10)–(12) correspond to the 1st, the 2nd, and the 3rd convolutional block of the MultiRes Block illustrated in Figure 4. The term $X_{c}$ in Equations (10)–(12) means the number of channels in an input X;
- MRB Equation (13) indicates MRB(X) is equal to the concatenation of the outputs of Equations (10)–(12) plus the input X. Concatenating (10) to (12) is taken as the result of MultiRes Block;
- The Equations (14) and (15) describe the residual path in Figure 4;
- The basic block of the residual path in Figure 4 described in Equation (14) is composed of the addition of the results of two convolutional layers of kernel sizes of 1 and 3;
- The four times repetition of the basic block of Equation (14) becomes the result of ResPath(RP) as described in Equation (15);
- Stages of MultiResUNet can be expressed in Equations (16)–(18);
- Equation (16) is for the 0th stage whose result is activated with ReLU after passing through the MRB;
- Equation (17) is for the encoder stage i (i = 1, 2, 3, 4), and the max pooled previous stage is activated with ReLU after passing through MRB;
- Equation (18) is for the decoder stage i (i = 5, 6, 7, 8) that concatenates the upsampled previous stage output with the corresponding encoder stage output at the same pooling level passed through the RP. The concatenation output is activated with ReLU after passing through the MRB.
  (10) $$m_{1}\left(X\right)=max\left(0,Conv^{3,1}{\left(X\right)}^{\frac{1}{6}X_{c}}\right)$$
  (11) $$m_{2}\left(X\right)=max\left(0,Conv^{3,1}{\left(m_{1}\left(X\right)\right)}^{\frac{2}{6}X_{c}}\right)$$
  (12) $$m_{3}\left(X\right)=max\left(0,Conv^{3,1}{\left(m_{2}\left(X\right)\right)}^{\frac{3}{6}X_{c}}\right)$$
  (13) $$MRB\left(X\right)=concat\left(m_{1}\left(X\right),m_{2}\left(X\right),m_{3}\left(X\right)\right)+X$$
  (14) $$f\left(X\right)=(max(0,Conv^{1,1}\left(X\right))+max(0,Conv^{3,1}\left(X\right)))$$
  (15) $$RP\left(X\right)=f^{4}\left(X\right)$$
  (16) $$Out_{0}^{stage}=max\left(0,MRB\left(Conv^{3,1}\left(I\right)\right)\right)$$
  (17) $$Out_{\left\{1,2,3,4\right\}}^{stage}=max\left(0,MRB(max^{2}\left(Out_{i-1}^{stage}\right)\right))$$
  (18) $$Out_{\left\{5,6,7,8\right\}}^{stage}=max\left(0,MRB\left(concat\left(RP(Out_{8-i}^{stage}),upsample^{2}\left(Out_{i-1}^{stage}\right)\right)\right)\right)$$

Equations (19)–(21) are used to describe the functionality of Half MultiResUNet:

- The stages of Half MultiResUNet can be expressed by Equations (19)–(21);
- Equations (19)–(21) are described similarly to Equations (16)–(18) of MultiResUNet. However, the number of stages is half. Equation (20) is for the encoder stage i (i = 1, 2) and Equation (21) for the decoder stage i (i = 3, 4).
  (19) $$Out_{0}^{stage}=max\left(0,MRB\left(Conv^{3,1}\left(I\right)\right)\right)$$
  (20) $$Out_{\left\{1,2\right\}}^{stage}=max\left(0,MRB(max^{2}\left(Out_{i-1}^{stage}\right)\right))$$
  (21) $$Out_{\left\{3,4\right\}}^{stage}=max\left(0,MRB\left(concat\left(RP(Out_{4-i}^{stage}),upsample^{2}\left(Out_{i-1}^{stage}\right)\right)\right)\right)$$

Equations (22)–(24) are used to describe the functionality of MultiResUNet.

- Stages of Quarter MultiResUNet can be represented in Equations (22)–(24);
- Equations (22)–(24) are similarly described as Equations (16)–(18) of MultiResUNet, but the number of stages is quarter. Equation (23) is for the encoder stage i (i = 1) and Equation (24) for the decoder stage i (i = 2).
  (22) $$Out_{0}^{stage}=max\left(0,MRB\left(Conv^{3,1}\left(I\right)\right)\right)$$
  (23) $$Out_{1}^{stage}=max\left(0,MRB(max^{2}\left(Out_{0}^{stage}\right)\right))$$
  (24) $$Out_{2}^{stage}=max\left(0,MRB\left(concat\left(RP(Out_{0}^{stage}),upsample^{2}\left(Out_{1}^{stage}\right)\right)\right)\right)$$

In the MultiResUNet, the size of the receptive fields can increase up to 38,000 px through many poolings and the convolutional layers with kernel size greater than 1, as shown in Figure 3 and as described in Equations (17) and (18). Considering that most of the information for accurate prediction is within 50 px, the network has unnecessarily large receptive fields, which can lead to excessive ineffective operations. However, the receptive field sizes of Half MultiResUNet and Quarter MultiResUNet are 200 px and 20 px, respectively. We expected that the receptive field size 200 px would reflect data features well, and the size 20 px would be a little insufficient for learning features.

## 4. Experiment

### 4.1. Dataset

**Figure 7:**
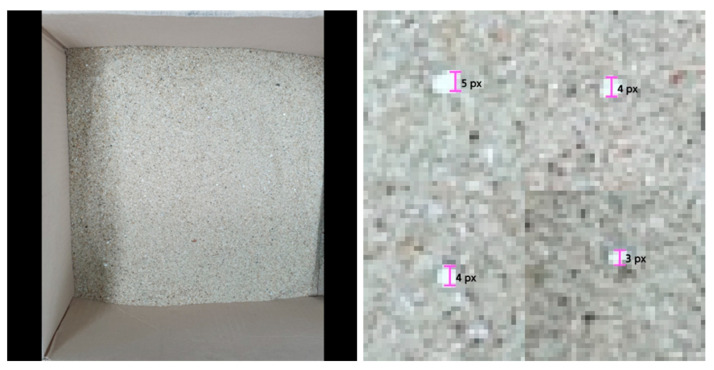
Original sand photo and a photo to show the size of microplastics.

Our dataset consisted of photos of sand, mixed with microplastic particles. The original size of the photos is 1440 × 1440, as shown in the left photo of Figure 7. However, the size of the photos in the dataset was 512 × 512. These were generated by cropping the original photo into 512 × 512 images with 100 pixel boundaries, overlapped with adjacent photos. The size of a foreground object, that is, microplastic, is usually 3–10 pixels, as shown in the right photo of Figure 7.

**Figure 8:**
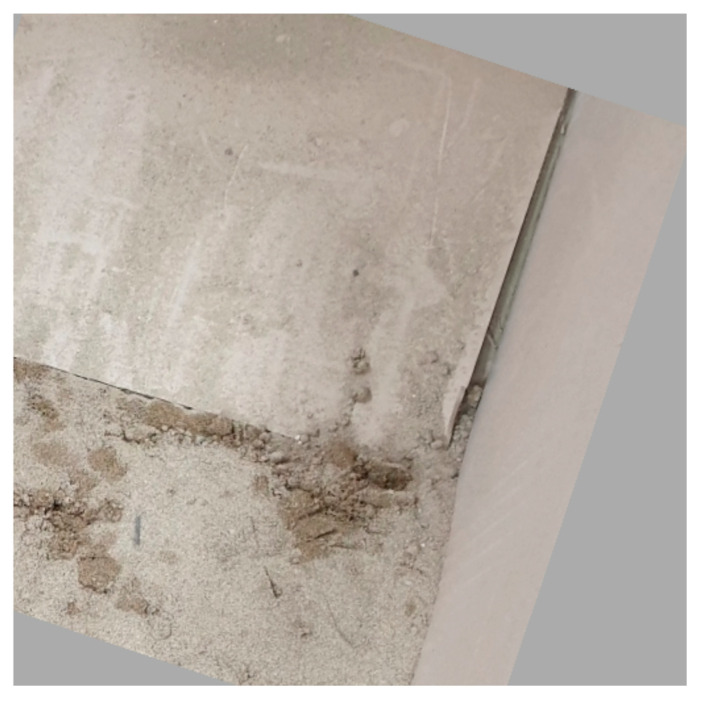
Photo generated through a 45° random rotation for data augmentation, during training.

As shown in Figure 8, during training, we applied data augmentation, such as 20% random shifting, 45° random rotation [23], and random flip for the images in the dataset. The empty space caused by shifting or rotation was filled with the mean pixel value [24] of all the photos in the dataset.

### 4.2. Loss Function

The dataset photos contain only a few minuscule microplastics. Therefore, false or background pixels in a photo usually occupy more than 99% of the pixels when calculated, that is, it is a highly imbalanced classification problem. Hence, we used the weighted binary cross entropy [25] loss function.

Equations (25)–(29) are used to describe the weighted binary cross entropy.

- In the Equations (25)–(29), Y is the batch of the ground truth set and $\hat{Y}$ is the batch of the data predicted by the neural network. One specific ground truth (prediction) image in the batch $\hat{Y}$ is expressed in ${\hat{Y}}_{i}$;
- The weights for both classes, true (microplastic) and false (background), assigned to reflect the class imbalance for the training images with 512 × 512 resolution are shown in Equations (25) and (26), respectively;
- The original cross-entropy loss formula is given in Equation (27);
- To accommodate the class imbalance, we multiplied the formula ‘WeightMat’ shown in Equation (28), with the CrossEntropy Equation (27);
- The final loss function with batch size ‘*n*’ is given by Equation (29).
  (25) $$w_{T}=\frac{512\times 512}{\sum Y_{i}}$$
  (26) $$w_{F}=\frac{512\times 512}{512\times 512-\sum Y_{i}}$$
  (27) $$CrossEntropy\left(Y_{i},\;{\hat{Y}}_{i}\right)=-\left(Y_{i}\log{\hat{Y}}_{i}+\left(1-Y_{i}\right)\log\left(1-{\hat{Y}}_{i}\;\right)\right)$$
  (28) $$WeightMat=Y_{i}w_{T}+\left(1-Y_{i}\right)w_{F}$$
  (29) $$Loss=\frac{1}{n}\overset{n}{\underset{i=1}{\sum }}\;\sum \left(CrossEntropy\left(Y_{i},\;{\hat{Y}}_{i}\right)\circ WeightMat\right)$$

### 4.3. Training and Validation

The Adam [26] optimizer was employed during training. The parameter Beta1 was set to 0.9, Beta2 to 0.999, and Epsilon to 1e-7. Beta1 and Beta2 are momentum hyper-parameters used to adjust the gradient decay rate. Epsilon is a value used to avoid the division by zero during gradient calculation. The exponential decay schedule [27] was applied to the learning rate. The rate started from 1e-3 and decreased by 4% every 800 steps. Each model was trained for 150 epochs using the above settings.

Five-fold cross validation was applied to obtain the average recall weighted (r.w.) F1 score and r.w. mIoU for all five NNs. In k-fold cross validation, the entire dataset is divided into K partitions, different 1/K partitions are used as test sets, and the remaining partitions are used as training sets. So, K different combinations of test and training sets are used. The final segmentation result for each pixel was considered to be true when its corresponding sigmoid output value was greater than 0.5.

The dataset consisted of 6176 photos. For five-fold cross validation [28], 20% of the data were used for the test data and the remaining 72% was used as training data with 8% as the validation data.

### 4.4. Segmentation Performance Comparison and Analysis

**Figure 9:**
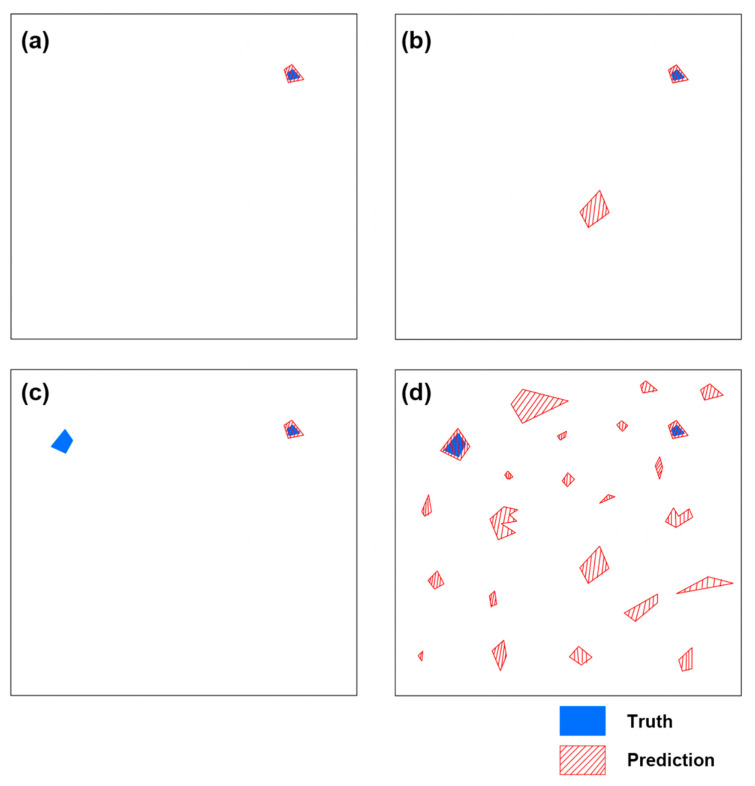
Hypothetical prediction result. (**a**) The best result. (**b**) The result showing insufficient precision. (**c**) The result showing insufficient recall. (**d**) The result showing excessively low precision.

In the process of sorting out microplastics mixed with sand in the real world, it is important to filter out all possible microplastic. In other words, it is more important for the neural network model to predict all the truths. A metric satisfying this condition can be recall, which is a metric representing the percentage of truth restored through model prediction from the entire truth, and recall can be expressed in Equation (30). The case shown in Figure 9b is the case where recall is 100%. Although there are results of sand particles mis-predicted as plastic, all plastics can be found. Conversely, the case as shown in Figure 9c, is the case where recall is 50%. There were no mis-predicted sand particles as plastic at all, but only half of the plastic was found.

However, as shown in Figure 9d, if the proportion of sand particles is excessively higher than that of microplastic, it will be difficult to determine whether they have been filtered well or not. So, the precision expressed by Equation (31) should also be an important metric. Therefore, if recall is at a satisfactory level, it can be said that the higher the precision, the better the model. In other words, if the predicted particles contain all plastics but most of them are sand particles, it is hard to say that the model is an efficient model. Therefore, as shown in Figure 9a, if the plastic restoration rate, i.e., recall, is high, the model with a high plastic composition ratio in the particles predicted as microplastic, i.e., high precision, has high efficiency.

In the case of Figure 9a, both recall and precision are 100%, and in the case of Figure 9b, recall is 100%, but precision is 50%. In the case of Figure 9c, recall is 50% and precision is 50%, and in the case of Figure 9d, recall is 100% but precision is less than 10%.

(30) $$Recall=\frac{TP}{TP+FN}$$

(31) $$Precision=\frac{TP}{TP+FP}$$

Table 1 shows the performance of each neural network model with two metrics, recall and precision. For the second case of MultiResUNet, the recall is 94%, it can be seen that all microplastics are well extracted or predicted. However, when the precision is 1.9%, it can be seen that the FP, which incorrectly predicts sand as microplastic, is too high. In other words, 94% of all the microplastics are detected as microplastic. However, it is difficult to say that the model is practical because more than 98% of the particles predicted as microplastic are sand. Therefore, the measurement of the model performance needs to be based on the multiplication of recall and precision. The metric F1 score that appears next may be one of such a metric since it is based on recall x precision.

F1 score [29] and mIoU [30,31] metrics were used for comparing the segmentation performances of the five NNs. Equations (32) and (33) show the formulas for the two metrics. Recall and precision are both necessary metrics for model evaluation, but either one alone is not sufficient to evaluate a neural network model. Therefore, we evaluate the performance of the neural network model through the F1 score, which can consider both recall and precision, and the mIoU, which considers only TP, FP, and FN, excluding excessively high TN. F1 score is a harmonic mean of two metrics so that precision and recall can be balanced in one metric. The metric mIoU can be said to be a form of adding FN to the denominator of the precision equation or adding FP to the denominator of the recall equation. For example, even if the recall of a model is 1, if its FP for mispredicting sand as microplastic increases, its mIoU decreases that much. Therefore, the metric mIoU can increase only when both precision and recall increase.

When the recall of a model is 1 and its precision is 0.25 or more, all microplastics were found and the microplastic composition ratio in the particles predicted as microplastic is 1/4. We consider that the model has minimal performance. In other words, for a given neural network model to have the least practicality, its prediction needs to include 100% of all microplastics, and more than 25% among those predicted as microplastics should be microplastics. This is our subjective judgment criterion for a practical model. In that case, the F1 score of the model is 0.4 that is our minimum criterion for the usefulness of the neural network model. In addition, the mIoU of a model can also be said to have least effective performance when the precision of the model is 25% or more and its recall is 1. So, our minimum performance criterion of a model in the metric mIoU is 0.25.

(32) $$F1\;Score=2\frac{Precision\;\cdot \;Recall}{Precision+Recall}$$

(33) $$mIoU=\frac{TP}{TP+FP+FN}$$

Table 2 shows the F1 score and mIoU of each network for the five cases of five-fold cross validation. Quarter MultiResUNet, Half U-net, and MultiResUNet appeared to face difficulty in converging the weights, that is, in training. We denoted the maximum F1 score and mIoU with bold numbers, among the five cases. As shown in Table 2, Half MultiResUNet and Quarter MultiResUNet showed relatively low variations in F1 score and mIoU, among the five cases than the prior three networks.

As shown in Table 2, U-net obtained the lowest average F1 score and mIoU. Half U-net showcased a slightly better average performance than U-net, whereas MultiResUNet showed a slightly better average performance than Half U-net. The best average performance was seen in Half MultiResUNet. Quarter MultiResUNet exhibited a worse average performance than Half MultiResUNet. According to the experiment using Quarter MultiResUNet, it appeared that the single encoder MultiRes block and the corresponding decoder MultiRes block may not be sufficient to achieve better segmentation performance than Half MultiResUNet.

Half MultiResUNet can be said to be a model that satisfies the minimum criteria presented above. However, it is necessary to further consider whether the F1 score and mIoU are suitable metrics for our purpose. In fact, for us, detecting all plastics (Recall) is more important than reducing the proportion of sand in the particles predicted as microplastics (Precision). However, F1 score and mIoU are metrics that consider recall and precision in equal weight. For example, recall 100%, precision 25%, and recall 40%, precision 40% are the same since F1 score of both is the same 40%. However, in the latter case, since the recall is 40%, i.e., only 40% of the microplastics are detected, and the precision is also 40%, the performance is very poor from our point of view of the detection and removal of microplastics. To prevent such problems, we propose to use recall-weighted (r.w.) F1 score and r.w. mIoU that gives importance to recall. Two metrics are described in Equations (34) and (35). In terms of r.w. F1 score, the model with recall 100% and precision 25% has 40% r.w. F1 score, but the model with recall 40% and precision 40% has only 16% of r.w. F1 score.

Since we set the criteria of adequate performance for the minimum practical model as recall 100% and precision 25%, the minimum performance criteria for r.w. F1 score and r.w. mIoU should still be at least 40% and 25%, respectively. Table 3 is made based on our proposed metrics r.w. F1 score and r.w. mIoU. As shown in Table 3, only Half MultiResUNet satisfies such minimum requirements for the neural network models.

(34) $$Recall-weighted\;F1\;Score=2\frac{Precision\;\cdot \;Recall}{Precision+Recall}*Recall$$

(35) $$Recall-weighted\;mIoU=\frac{TP}{TP+FP+FN}*Recall$$

Table 4 summarizes the required number of floating point operations (FLOPs), and the number of parameters for the five NNs. The ‘FLOPs’ means the number of floating additions and multiplications required for the prediction with one input data, and the ‘Parameters’ means the number of weights or variables included in a model. Units “B” and “M” mean billions and millions, respectively. As shown in Table 4, U-net, Half U-net, and MultiResUNet need a considerably high number of FLOPs and a number of parameters. Contrastingly, Half MultiResUNet and Quarter MultiResUNet need a fairly low number of FLOPs, a small number of parameters, and have better segmentation performance. In other words, Half MultiResUNet and Quarter MultiResUNet are computationally efficient as well as efficient in performance.

**Figure 10:**
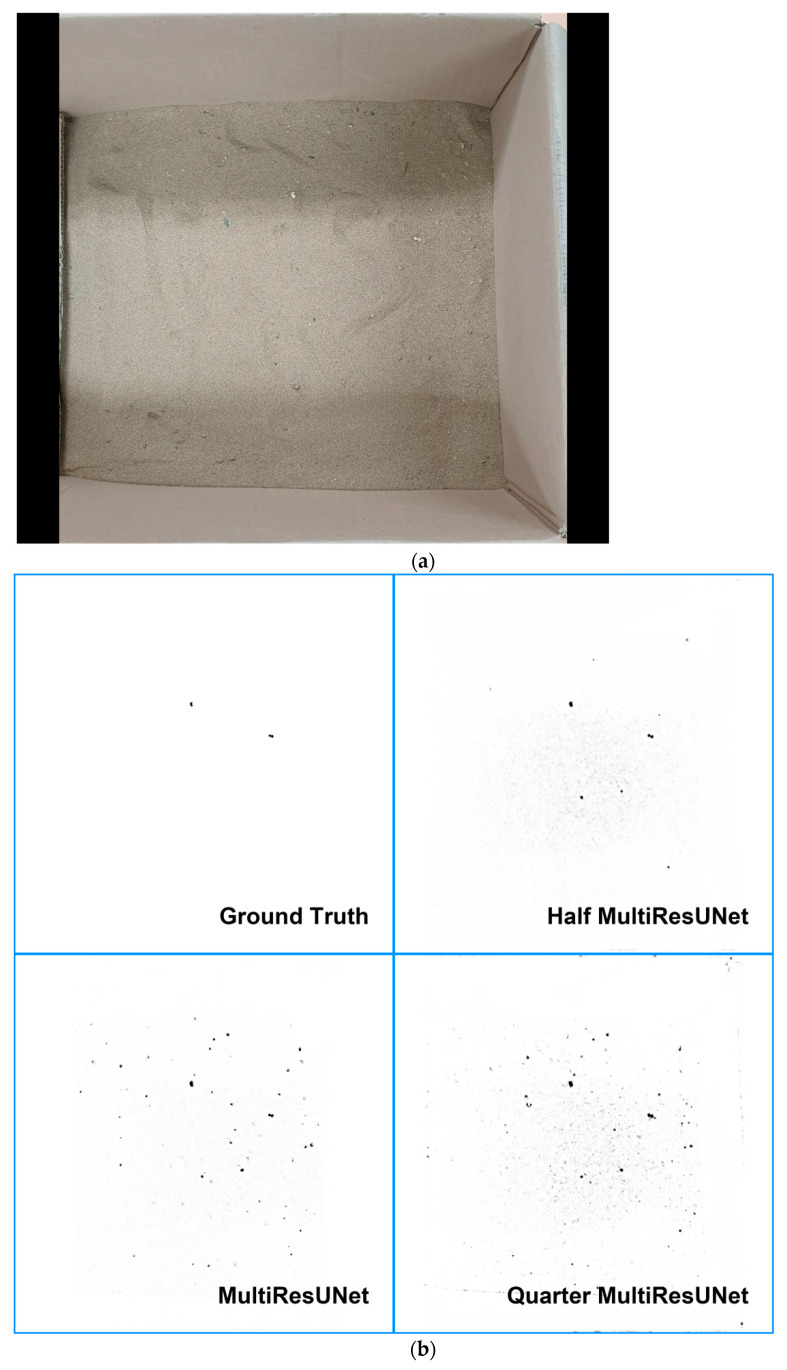
Prediction result images by 3 models with source image (**a**) and ground truth image. (**a**) Source Image from one of test dataset. (**b**) Ground truth and outputs of 3 models from one of test dataset.

Figure 10a is one of the test set images. Figure 10b shows the ground truth image and the prediction images by the corresponding model given the input image of Figure 10a. As shown in Figure 10b, all models seem to have found almost all microplastics, i.e., recall is nearly 1. However, Half MultiResUNet has relatively lower FP over the other models, i.e., a smaller number of sand particles incorrectly predicted as microplastics, and it can be applicable to actual problems.

### 4.5. Observations on Weight Histograms of U-Net and MultiResUNet

**Figure 11:**
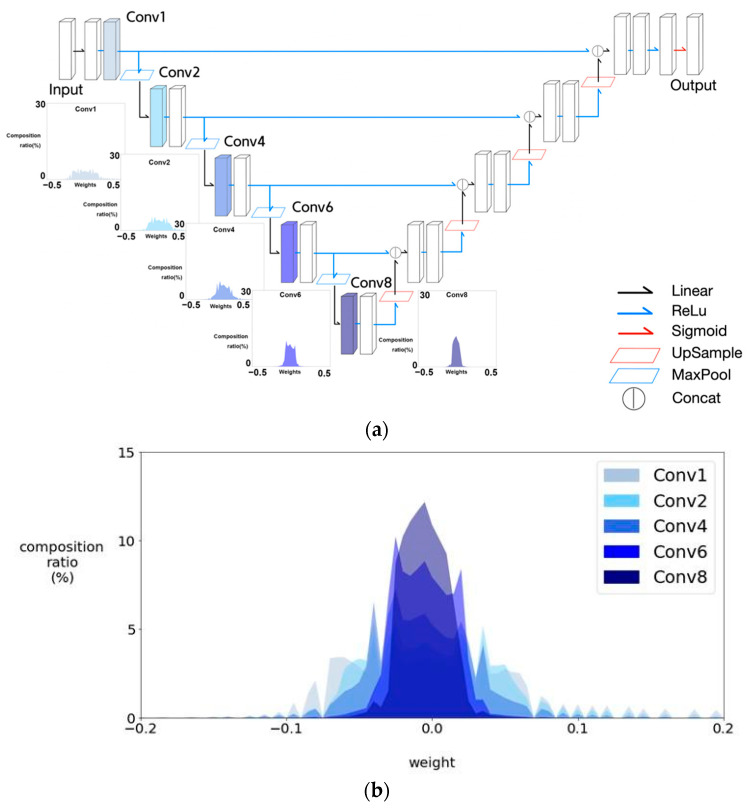
Kernel weights histograms of several encoder convolutional layers of U-net visualized using TensorBoard. (**a**) Weight histograms of specified convolutional layers with colors in U-net. (**b**) Overlapped weight histograms of specified convolutional layers.

Observations on kernel weight histograms in this section come from the maximum r.w. F1 score cases in Table 2. We observed that the kernel weight histograms of U-net visualized using TensorBoard, showed that kernel weights approached zero as the encoder stage proceeded, as shown in Figure 11. In other words, the latter half of the encoder stages were not significantly involved in feature learning. Hence, we added Half U-net as a candidate network.

**Figure 12:**
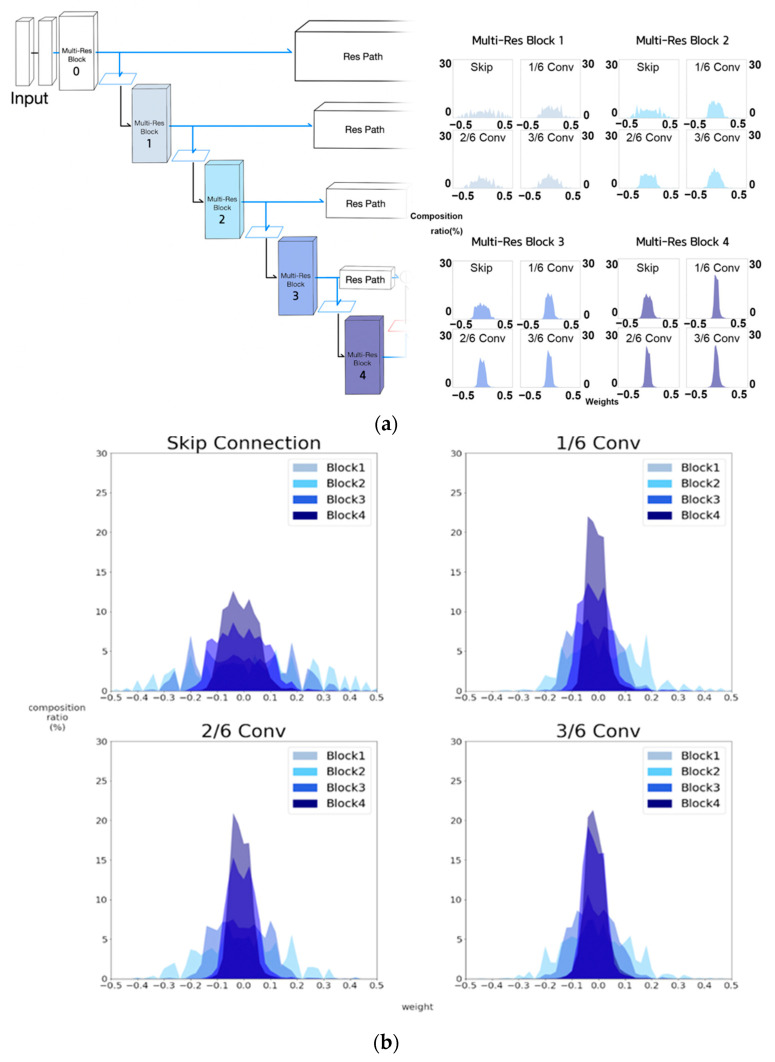
Weight histograms of MultiRes blocks of MultiResUNet. (**a**) Weight histograms of layers within MultiRes blocks 1, 2, 3, and 4. (**b**) Overlapped weight histograms for each component of MultiRes blocks 1, 2, 3, and 4.

In the case of MultiResUNet, however, we noticed that although the encoder stage proceeded, the convergence of kernel weights closes to zero did not appear to be obvious, as shown in Figure 12, likely by virtue of the MultiRes block and Res path. However, as the encoder stage proceeded, we observed that within the MultiRes block, the kernels of feature extraction connections are less activated than the kernels of skip connections, as shown in the lower half of Figure 12. In other words, kernel weights of non-skip connection layers of MultiRes blocks 3 and 4, appeared to approach zero, that is, such layers were not used for feature learning. As a result, we deemed it necessary to explore Half MultiResUNet and quarter MultiResUNet, as candidate networks.

### 4.6. Observations on Weight Histograms of Half and Quarter MultiResUNet

**Figure 13:**
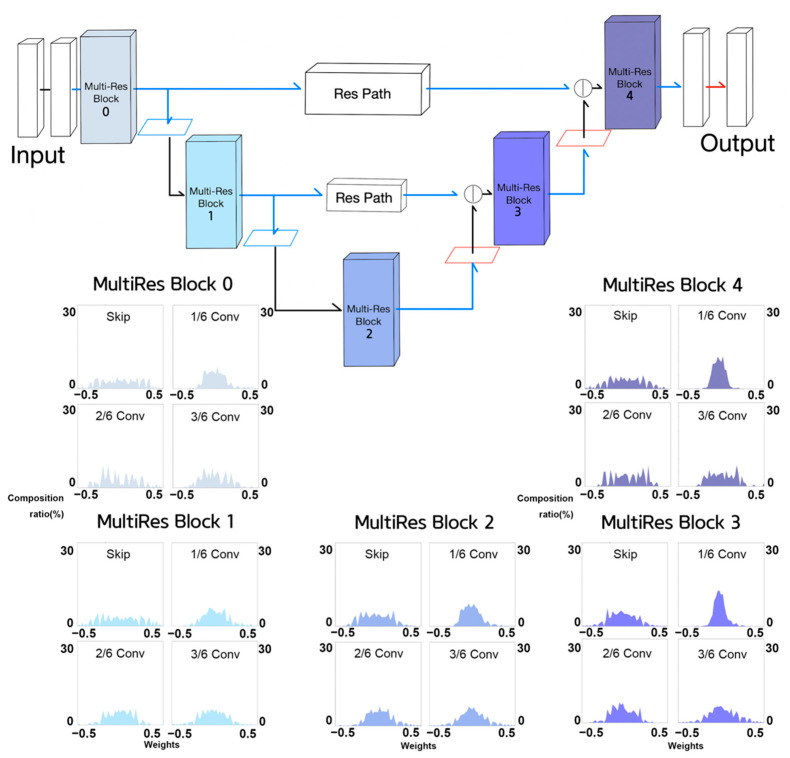
Weight histograms of each component of MultiRes blocks in Half MultiResUNet.

Figure 13 shows the weight histograms of each component of the MultiRes blocks of Half MultiResUNet. Kernel weights are generally widely distributed over a given range. In other words, most components of the MultiRes blocks appeared to be involved in feature learning. This may have contributed to the leading segment performance of Half MultiResUNet, among the five NNs. Some components of MultiRes blocks 3 and 4 display weight distributions centered around zero. Hence, we performed experiments using Quarter MultiResUNet.

**Figure 14:**
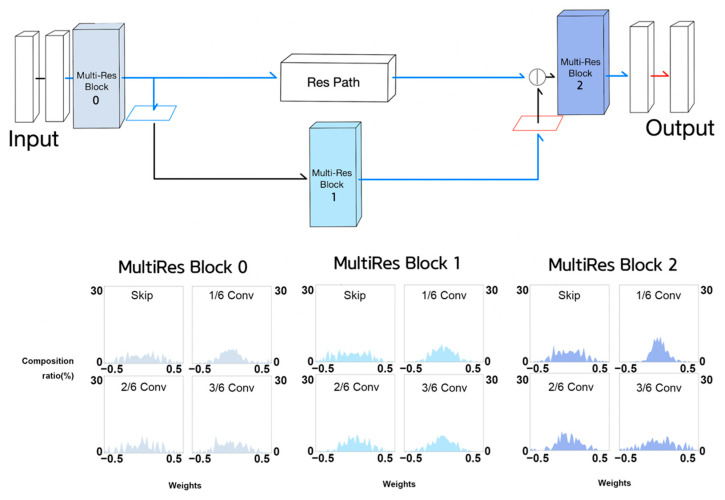
Quarter MultiResUNet of kernel weights histogram.

Figure 14 shows the weight histograms of each component of MultiRes blocks 1, 2, and 3 in Quarter MultiResUNet. As shown in Figure 14, none of the components of the MultiRes blocks are centered close to zero. This implies that all the components were involved in feature learning. As shown in Table 2, Quarter MultiResUNet experienced a slightly worse performance than Half MultiResUNet. This may have been a result of the absence of the second encoder MultiRes block, and its corresponding decoder MultiRes block, as in Half MultiResUNet. We speculate that these blocks may be involved in the learning of somewhat larger features, as compared to the first and last block pairs. Another probable cause may be that Quarter MultiResUNet does not have a sufficient number of channels for feature learning in MultiRes blocks, as compared to Half MultiResUNet. However, we observed that Quarter MultiResUNet with four times as many channels did not lead to a better performance. Therefore, the major reason for the inferior performance of Quarter MultiResUNet over Half MultiResUNet was determined to be the absence of the second encoder and decoder MultiRes block pair.

## 5. Conclusions

Microplastics are tiny objects with no regular shape. To find an appropriate NN for microplastic segmentation, we explored two existing NNs: U-net and MultiResUNet. Our observation of kernel weight histograms indicated that the later encoder stages of the two networks are not useful for capturing small features. Therefore, we derived reduced versions of U-net and MultiResUNet, such as Half U-net, Half MultiResUNet, and Quarter MultiResUNet. Experiments showed that Half MultiResUNet displayed the best average r.w. F1 score and r.w. mIoU and Quarter MultiResUNet the second best average r.w. F1 score and r.w. mIoU for our microplastic dataset. They also require very low floating point operations and much lower number of parameters over U-net and MultiResUNet. Hence, they may be suitable for use in embedded applications.

We may summarize the neural networks explored in this paper as follows. Existing U-net and MultiResUNet have unnecessary layers for microplastic segmentation and rather large computational cost though they may be useful for the segmentation of middle or large sized objects. Half U-net derived from U-net has very low microplastic segmentation performance though it has relatively low number of parameters. Half MultiResUNet and Quarter MultiResUNet derived from MultiResUNet have good segmentation performance and very low computational cost. However, they will not show good segmentation performance for middle or large sized objects since they have limited size in receptive fields.

Our observations on kernel weight histograms help in finding reduced neural network architectures. Kernel weight histogram makes it easier to determine the degree of contribution of the layer and to find the improvement direction of the model so that we can delete the layer or increase the embedding level. However, if the weight does not completely converge to 0, it is difficult to determine whether the layer contributes. In addition, the achieved 40% r.w. F1 score should be improved by further exploration of neural network architectures for efficient applications. Improved versions of neural networks for tiny object segmentation are expected to be used not only in microplastics segmentation, but also in finding victims from drone images taken on the mountain and in segmenting objects in satellite images.

## Figures and Tables

**Table 1:** Precision and recall of five neural networks.

**Model** **(150 Eps)**	**1st Case**		**2nd Case**		**3rd Case**	
	**Precision**	**Recall**	**Precision**	**Recall**	**Precision**	**Recall**
U-net	0	0	0.0692	0.9644	0	0
Half U-net	0.0565	0.9370	0.0437	0.9470	0	0
MultiResUNet	0.1583	0.9386	0.0190	0.9448	0.0329	0.9557
Half MultiResUNet	0.2899	0.9180	0.3175	0.9322	0.2771	0.9404
Quarter MultiResUNet	0.1065	0.9517	0.1451	0.9772	0.1828	0.9624
**Model** **(150 Eps)**	**4th Case**		**5th Case**		**Average**	
	**Precision**	**Recall**	**Precision**	**Recall**	**Precision**	**Recall**
U-net	0.0178	0.8138	0	0	0.0174	0.3556
Half U-net	0.0357	0.8547	0	0	0.0272	0.5478
MultiResUNet	0.0092	0.8813	0.0138	0.8972	0.0466	0.9235
Half MultiResUNet	0.2828	0.9447	0.2219	0.9832	0.2778	0.9437
Quarter MultiResUNet	0.2214	0.9456	0.1316	0.9817	0.1575	0.9637

**Table 2:** F1 score and mIoU of five neural networks.

**Model** **(150 Eps)**	**1st Case**		**2nd Case**		**3rd Case**	
	**F1 Score**	**mIoU**	**F1 Score**	**mIoU**	**F1 Score**	**mIoU**
U-net	0	0	0.1291	0.0690	0	0
Half U-net	0.1067	0.0563	0.0836	0.0436	0	0
MultiResUNet	0.2710	0.1567	0.0372	0.0189	0.0636	0.0328
Half MultiResUNet	0.4407	0.2826	0.4737	0.3103	0.4281	0.2723
Quarter MultiResUNet	0.1916	0.1060	0.2526	0.1446	0.3072	0.1815
**Model** **(150 Eps)**	**4th Case**		**5th Case**		**AVERAGE**	
	**F1 Score**	**mIoU**	**F1 Score**	**mIoU**	**F1 Score**	**mIoU**
U-net	0.0349	0.0178	0	0	0.0328	0.0173
Half U-net	0.0685	0.0355	0	0	0.0517	0.0270
MultiResUNet	0.0181	0.0091	0.0272	0.0138	0.0834	0.0462
Half MultiResUNet	0.4352	0.2782	0.3621	0.2210	0.4279	0.2728
Quarter MultiResUNet	0.3588	0.2186	0.2321	0.1313	0.2685	0.1563

**Table 3:** r.w. F1 score and r.w. mIoU of five neural networks.

**Model** **(150 Eps)**	**1st Case**		**2nd Case**		**3rd Case**	
	**r.w. F1 Score**	**r.w. mIoU**	**r.w. F1 Score**	**r.w.** **mIoU**	**r.w. F1 Score**	**r.w. mIoU**
U-net	0	0	0.1245	0.0665	0	0
Half U-net	0.0999	0.0528	0.0792	0.0413	0	0
MultiResUNet	0.2544	0.1471	0.0351	0.0179	0.0608	0.0313
Half MultiResUNet	0.4046	0.2594	0.4416	0.2893	0.4026	0.2561
Quarter MultiResUNet	0.1823	0.1009	0.2468	0.1413	0.2956	0.1747
**Model** **(150 Eps)**	**4th Case**		**5th Case**		**Average**	
	**r.w. F1 Score**	**r.w. mIoU**	**r.w. F1 Score**	**r.w. mIoU**	**r.w. F1 Score**	**r.w. mIoU**
U-net	0.0284	0.0145	0	0	0.0306	0.0162
Half U-net	0.0585	0.0303	0	0	0.0475	0.0249
MultiResUNet	0.0159	0.0080	0.0244	0.0124	0.0781	0.0433
Half MultiResUNet	0.4111	0.2628	0.3560	0.2173	0.4032	0.2570
Quarter MultiResUNet	0.3393	0.2067	0.2279	0.1289	0.2584	0.1505

**Table 4:** Required FLOPs and the number of parameters for five NNs.

Model (Input Size)	FLOPs	Parameters
U-net (512 × 512)	329.7 B	21.9776 M
Half U-net (512 × 512)	204.0 B	1.7724 M
MultiResUNet (512 × 512)	204.8 B	13.5469 M
Half MultiResUNet (512 × 512)	42.9 B	0.2149 M
Quarter MultiResUNet (512 × 512)	21.9 B	0.0445 M

## Data Availability

Not applicable.
